# Solitary 15 cm splenic abscess successfully treated with percutaneous drainage

**DOI:** 10.1016/j.idcr.2022.e01413

**Published:** 2022-01-25

**Authors:** Toshiaki Tsurui, Alan T. Lefor, Kauzhiro Nishida

**Affiliations:** a Tokyo Bay Urayasu Ichikawa Iryo Center, Tokyo, Japan; b Jichi Medical University, Tochigi, Japan

**Keywords:** Splenic abscess, Percutaneous drainage

## Abstract

Splenic abscesses are rare, but can be life-threatening. Antibiotics, percutaneous drainage and splenectomy are the usual treatment options. However, there is no ideal algorithm for choosing among these options. A man in his 60 s presented with 10 days of left upper quadrant pain and abdominal distension. Computed tomography (CT) scan of the abdomen revealed a splenic abscess measuring 15 cm in diameter. Transesophageal echocardiography confirmed the diagnosis of infectious endocarditis. Ultrasound-guided percutaneous drainage was performed and *Streptococcus anginosus* grew in cultures of both blood and intrasplenic fluid. The patient was treated with intravenous antibiotics and continuous drainage for 8 weeks. The abscess cavity nearly disappeared on follow-up CT scan. Percutaneous drainage should be considered for a solitary unilocular splenic abscess even if the abscess is large.

## Introduction

Splenic abscesses are relatively uncommon, and are typically associated with various sites of infection. The incidence of splenic abscess is estimated to be from 0.2% to 0.7% in autopsy series [1]. The mortality rate is still over 10% even with current diagnostic modalities and appropriate treatment [2], [3]. Management includes antibiotics and source control, which include splenectomy and percutaneous drainage. Splenectomy has been considered the definitive treatment for splenic abscess. Percutaneous drainage has been increasingly chosen as an alternative treatment. Several indications were suggested for percutaneous drainage. However, no consensus has been established. It is important to clarify the indications for the various treatment modalities. This report of a patient with a large splenic abscess successfully treated with percutaneous drainage will provide a further insight into the indications for percutaneous drainage.

## Case presentation

A man in his 60 s presented with 10 days of left upper quadrant pain and abdominal distension. His past medical history was significant for hypertension, type 2 diabetes mellitus, dyslipidemia and atrial fibrillation. He had smoked one pack of cigarettes daily for 45 years and did not drink alcohol regularly. He presented with tachycardia (135 beats per minute) and was afebrile. Physical examination revealed a flat and soft abdomen with mild tenderness in the left upper quadrant. His oral hygiene was poor with extensive dental caries.

**Fig. 1:**
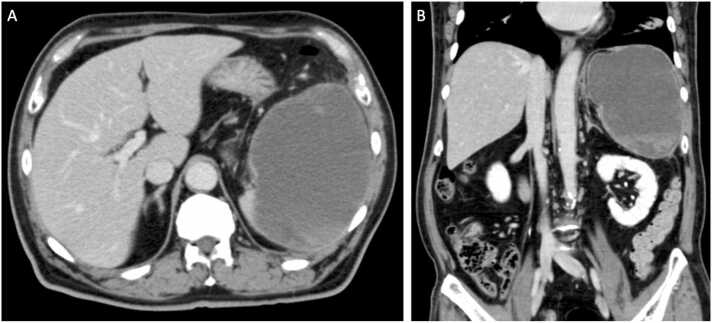
Contrast-enhanced CT of the abdomen on admission. It demonstrates a solitary hypodense lesion measuring 15 × 10 cm. Splenic capsule is thinned around the entire circumference.

Laboratory studies showed an elevated leukocyte count (10,600 per microliter) and C-reactive protein (17.84 mg/dL). Ultrasonography of the abdomen showed a single hypoechoic intrasplenic lesion and contrast-enhanced CT scan demonstrated a solitary hypodense lesion measuring 15 × 10 cm without internal septations (Fig. 1). The splenic capsule was thinned around its entire circumference. A splenic abscess was clinically suspected and blood cultures were obtained. Transesophageal echocardiography showed a mobile vegetation on the aortic valve, which confirmed the diagnosis of infectious endocarditis.

Ampicillin-sulbactam was empirically started. He underwent ultrasound-guided percutaneous drainage of the splenic abscess which revealed brown purulent fluid. *Streptococcus anginosus* grew in cultures from both blood and the drainage. Ampicillin-sulbactam was changed to benzylpenicillin based on antibiotic susceptibility.

**Fig. 2:**
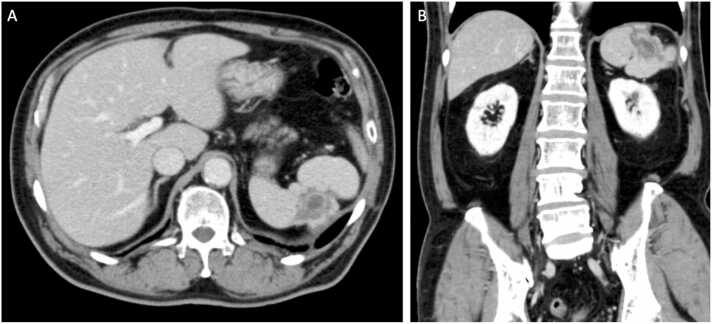
Follow-up CT scan performed 4 months after the discharge. Abscess cavity almost completely disappeared.

Four weeks after admission, the splenic abscess was reduced in size but still present on CT scan. Therapy was changed to oral amoxicillin after an 8-week course of intravenous antibiotic therapy and the patient was discharged home. He remained asymptomatic at follow-up 1 week after discharge, and antibiotic therapy was discontinued. Follow-up CT scan performed 4 months later showed almost complete resolution of the splenic abscess (Fig. 2).

## Discussion

Splenic abscesses often occur in patients with disseminated infectious foci, which accounts for about half of the cases. The leading cause of infection is infective endocarditis. Splenic abscesses complicated infective endocarditis in 10–20% of patients [2], [4], [5] while 5–10% of patients with infective endocarditis also have a splenic abscess in autopsy series [6]. Contiguous spread of infection and accidental or iatrogenic trauma to the spleen can lead to splenic abscesses in a minority of patients [2]. The most important risk factor is an immunodeficient state, resulting from various causes such as chemotherapy, steroid use, hematologic malignancies or acquired immunodeficiency syndrome. Reportedly 15–34% of patients with splenic abscess were immunocompromised [2], [3]. The present patient was found to have a splenic abscess as the first manifestation of infectious endocarditis. He may have been immunocompromised due to diabetes mellitus but this was not documented and remains speculative.

The number and size of abscesses vary depending on the report. Single (solitary) abscesses were seen in 50–70% of splenic abscesses and multiple abscess in 30–50% [2], [3], [4], [7], [8], [9]. while 60–90% of abscesses are loculated and have internal septations [2], [9], [10]. The reported mean size of abscesses ranged from 4 cm to 6 cm [3], [10].

Traditionally, antibiotics and splenectomy have been the gold standard of treatment for patients with a splenic abscess. Percutaneous drainage of intra-abdominal abscesses is widely used.It is considered to be a safe and effective technique for both diagnosis and treatment though potential complications of fistula formation, pneumothorax, and bowel perforation are reported [11]. However, reports of splenic abscess treated with drainage alone are limited. This is due to a low incidence of splenic abscess and the risk of the procedure, which is attributed to the vascularity of the spleen and the difficulty in accessing the abscess [12]. Recently, with improvements in imaging modalities, more studies have been reported. The outcomes of percutaneous drainage for the treatment of splenic abscess in more recent reports is comparable to those for splenectomy [3], [8], [13]. One of the advantages of percutaneous drainage is preservation of the spleen, which avoids the complication of overwhelming postsplenetomy sepsis. Percutaneous drainage can also be performed in patients with elevated perioperative risk [14].

There are no well-accepted indications for percutaneous drainage. Some authors suggest that percutaneous drainage should be performed only for solitary and, unilocular or bilocular splenic abscesses [10], [13], [14]. The size of the abscess is also a determining factor in selecting appropriate management. Percutaneous drainage is indicated for abscesses larger than from 3 to 6 cm [7], [10], [15]. In contrast, smaller abscesses are treated with antibiotics and/or percutaneous needle aspiration due to the difficulty in placing a drain inside an abscess cavity. Splenectomy is reserved for patients who are clinically unstable, who failed to improve with percutaneous drainage, or who have multiple abscesses [14], [16]. However, some authors suggest that splenectomy should be performed for abscesses larger than 10 cm [15].

A patient with a 15 cm splenic abscess successfully treated with ultrasound-guided percutaneous drainage is reported. Percutaneous drainage was selected for treatment because the abscess was solitary as well as unilocular, and thinning of the splenic capsule was felt to have considerable risk for intraoperative rupture. The patient responded to percutaneous drainage and antibiotics, thereafter his abscess almost completely disappeared although it required an extended period of hospitalization. The outcome suggests that percutaneous drainage is a reasonable treatment option for patients with a splenic abscess even if the abscess is large. Several reports showed that abscess size does not correspond to the success rate for treatment of a splenic abscess [3], [9], [15]. To confirm the validity of the indications for percutaneous treatment, larger clinical trials are needed. The potential risks and benefits of each option must be tailored to the treatment for each patient in clinical decision-making.

## Conclusion

Splenic abscess is often associated with disseminated infections at sites elsewhere in the body. One of the most commonly associated conditions is infectious endocarditis. Although careful selection of these treatment options is the key to appropriate management, clear indications for each option have not been established yet. Percutaneous drainage should be considered when the splenic abscess is solitary and unilocular, and large enough (>3–5 cm) to drain even if the abscess exceeds 10 cm.

## Ethical approval

Not applicable.

## Funding

None.

## Consent

The patient provided verbal and written consent for publication.

## CRediT authorship contribution statement

Toshiaki Tsurui designed the case report and drafted the manuscript. Alan T. Lefor supervised and revised the manuscript. Kazuhiro Nishida conceived of and coordinated the study.

## Declaration of Competing Interest

The author report no declarations of interest.
